# Identification of Claudin-6 as a Molecular Biomarker in Pan-Cancer Through Multiple Omics Integrative Analysis

**DOI:** 10.3389/fcell.2021.726656

**Published:** 2021-08-02

**Authors:** Chiyuan Zhang, Cuishan Guo, Yan Li, Kuiran Liu, Qi Zhao, Ling Ouyang

**Affiliations:** ^1^Department of Obstetrics and Gynecology, Shengjing Hospital of China Medical University, Shenyang, China; ^2^School of Computer Science and Software Engineering, University of Science and Technology Liaoning, Anshan, China

**Keywords:** Cldn-6, pan-cancer analysis, prognostic biomarker, molecular biomarker, omics integrative analysis

## Abstract

Claudin-6 (CLDN6) is one of the 27 family members of claudins and majorly involved in the tight junction and cell-to-cell adhesion of epithelial cell sheets, playing a significant role in cancer initiation and progression. To provide a more systematic and comprehensive dimension of identifying the diverse significance of CLDN6 in a variety of malignant tumors, we explored CLDN6 through multiple omics data integrative analysis, including gene expression level in pan-cancer and comparison of CLDN6 expression in different molecular subtypes and immune subtypes of pan-cancer, targeted protein, biological functions, molecular signatures, diagnostic value, and prognostic value in pan-cancer. Furthermore, we focused on uterine corpus endometrial carcinoma (UCEC) and further investigated CLDN6 from the perspective of the correlations with clinical characteristics, prognosis in different clinical subgroups, co-expression genes, and differentially expressed genes (DEGs), basing on discussing the validation of its established monoclonal antibody by immunohistochemical staining and semi-quantification reported in the previous study. As a result, CLDN6 expression differs significantly not only in most cancers but also in different molecular and immune subtypes of cancers. Besides, high accuracy in predicting cancers and notable correlations with prognosis of certain cancers suggest that CLDN6 might be a potential diagnostic and prognostic biomarker of cancers. Additionally, CLDN6 is identified to be significantly correlated with age, stage, weight, histological type, histologic grade, and menopause status in UCEC. Moreover, CLDN6 high expression can lead to a worse overall survival (OS), disease-specific survival (DSS), and progression-free interval (PFI) in UCEC, especially in different clinical subgroups of UCEC. Taken together, CLDN6 may be a remarkable molecular biomarker for diagnosis and prognosis in pan-cancer and an independent prognostic risk factor of UCEC, presenting to be a promising molecular target for cancer therapy.

## Introduction

Claudins consist of 27 members in mammals that are essential for the regulation of paracellular permeability and the maintenance of cell polarity, due to their significant roles of being key components in tight junctions, involving almost all vital physiological and pathological bioprocesses (Peter and Goodenough, 2004; Lal-Nag and Morin, 2009; Barmeyer et al., 2015; Tabaries and Siegel, 2017). In recent years, claudins have been recognized as crucial regulators in the initiation, progression, and metastasis of cancers, playing distinct roles in a variety of cancers according to their different patterns of tissue-dependent expression (Tabaries and Siegel, 2017).

Claudin-6 (CLDN6) is a member of the claudin family and serves as a tight junction molecule, which plays a vital role in cell-to-cell adhesion in epithelial or endothelial cell sheets. It encodes the tetraspan membrane protein, with the size of 220 amino acids and molecular mass of 23,292 Da. CLDN6 has been identified to be the origination of cell adhesion signaling taking part in the regulation of nuclear receptor activity through targeting molecules of the nuclear receptor superfamily and managing their gene expression (Sugimoto et al., 2019). In terms of its various characters in human cancers, CLDN6 may be a helpful positive marker for further identification of atypical teratoid/rhabdoid tumors (AT/RTs) for diagnosis and therapy (Birks et al., 2010). CLDN6 is also reported to be a possible single prognostic marker and promising therapeutic target for a subgroup of intestinal type gastric cancer (Kohmoto et al., 2020). However, over-expression of CLDN6 may suppress the progression of breast cancer, whereas DNA methylation of CLDN6 can downregulate its gene expression and promote migration and invasion (Liu Y. et al., 2016). Recently, CLDN6 has been identified to be an oncofetal cell surface antigen for chimeric antigen receptor (CAR)-T cell targeting, due to its aberrant activation and high protein expression for solid cancers, while silence for healthy tissues, respectively (Reinhard et al., 2020).

However, as the previous studies reported, CLDN6 may take diverse parts in different cancers, even in different subtypes of the same cancer, either for cancer promotion or for cancer suppression. The discrepancies between the dual roles of CLDN6 in human cancers may be due to the heterogeneity and complexity of tumors. In order to provide a more systematic and comprehensive insight of CLDN6, to the best of our knowledge, we are the first to explore the expression and biofunction of CLDN6 from the perspective of pan-cancer, focusing on its diagnostic and prognostic values, and find that CLDN6 is not only significantly upregulated in 20 types of human cancers but also differently expressed in different molecular subtypes of seven cancer types and different immune subtypes of nine cancer types. Additionally, CLDN6 has a high accuracy in predicting acute myeloid leukemia (LAML), testicular germ cell tumors (TGCT), ovarian serous cystadenocarcinoma (OV), and uterine carcinosarcoma (UCS) while having notable correlations with the overall survival (OS), disease-specific survival (DSS), and progression-free interval (PFI) of uterine corpus endometrial carcinoma (UCEC), adrenocortical carcinoma (ACC), bladder urothelial carcinoma (BLCA), and stomach adenocarcinoma (STAD). Next, we put emphasis on UCEC and identify CLDN6 as an independent risk factor for OS, DSS, and PFI in UCEC. Moreover, we further investigate the co-expression genes correlated with CLDN6 and the differentially expressed genes (DEGs) between CLDN6 high expression group and low expression group. Taken together, CLDN6 is a potential biomarker for diagnosis and prognosis in pan-cancer and a promising molecular target for UCEC.

## Materials and Methods

### Gene Expression Analysis

The RNA-seq data and relevant clinical data across 33 tumor types and normal tissues of 15,776 samples were downloaded from The Cancer Genome Atlas (TCGA) database and the Genotype-Tissue Expression (GTEx) database by UCSC XENA^1^. The data of tumor cell line were downloaded from the Cancer Cell Line Encyclopedia (CCLE) database^2^. R software v3.6.3 was used for statistical analysis, and the ggplot2 package was used for visualization. The Wilcoxon rank sum test detected two sets of data, and *p* < 0.05 was considered statistically significant (ns, *p* ≥ 0.05; ^∗^, *p* < 0.05; ^∗∗^, *p* < 0.01; ^∗∗∗^, *p* < 0.001) (Vivian et al., 2017).

### CLDN6 Expression in Molecular Subtypes and Immune Subtypes of Cancers

We explored the correlations between CLDN6 expression and molecular subtypes or immune subtypes in pan-cancer from the TISIDB database (Ru et al., 2019), which integrates multiple data types to assess tumor and immune system interaction. We also explored the correlations between CLDN6 expression and immunomodulators in pan-cancer from the TISIDB database.

### Protein–Protein Interaction Network Building

A total of 50 CLDN6-binding proteins were acquired from the STRING web^3^ by setting the following main parameters: minimum required interaction score [“medium confidence (0.400)”] and active interaction sources (“Experiments, Text mining, Databases”). Then, Cytoscape (version 3.7.2) was applied for visualization of protein–protein interaction (PPI) network.

### Gene Ontology and Kyoto Encyclopedia of Genes and Genomes Enrichment Analyses

The Gene Ontology (GO) and Kyoto Encyclopedia of Genes and Genomes (KEGG) enrichment analyses were conducted for 50 CLDN6-binding proteins using the ggplot2 package for visualization and the cluster Profiler package for statistical analysis (Subramanian et al., 2005; Yu et al., 2012).

### Diagnostic Value Analysis

The receiver operating characteristic (ROC) curve was used to assess the diagnostic value of CLDN6 in pan-cancer. The area value under the ROC curve is between 0.5 and 1. The closer the area under the curve (AUC) is (1), the better the diagnostic effect is. AUC in 0.5–0.7 has a low accuracy, AUC in 0.7–0.9 has a certain accuracy, and AUC above 0.9 has a high accuracy.

### Survival Prognosis Analysis

Kaplan–Meier plots were used to assess the relationship between CLDN6 expression and prognosis (OS, DSS, and PFI) of cancers. Moreover, we further investigated the associations between CLDN6 expression and prognosis (OS, DSS, and PFI) in different clinical subgroups of UCEC. The survminer package was used for visualization, and the survival package was used for statistical analysis. The Cox regression was used in the hypothesis test, and *p* < 0.05 is considered statistically significant.

### Associations Between CLDN6 Expression and Different Clinical Characteristics in UCEC

The box plots and tables were presented for CLDN6 expression levels of patients with different clinical characteristics in UCEC. The RNA-seq data and related clinical data in level 3 HTSeq-fragments per kilobase per million (FPKM) format were downloaded from TCGA database, converted to transcripts per million reads (TPM) format, and then analyzed after log2 conversion. The Wilcoxon rank sum test was used to detect two groups of data, and *p* < 0.05 was considered statistically significant (ns, *p* ≥ 0.05; ^∗^, *p* < 0.05; ^∗∗^, *p* < 0.01; ^∗∗∗^, *p* < 0.001).

### Univariate and Multivariate Cox Regression Analyses in UCEC

Univariate and multivariate Cox regression analyses of CLDN6 and clinical characteristics were performed to identify their prognostic values in OS, DSS, and PFI of UCEC. The survival package was used for statistical analysis.

### Co-expression Gene Analysis of CLDN6 in UCEC

We explored the top 50 co-expression genes positively and negatively correlated with CLDN6 expression in UCEC. The gene co-expression heatmaps were displayed using the stat package. We also showed the correlations between CLDN6 expression and top 10 genes expression in the heatmap using Pearson correlation coefficient.

### DEGs Between CLDN6 High Expression and Low Expression Groups in UCEC

We explored the DEGs between different CLDN6 expression groups (low expression group: 0–50%; high expression group: 50–100%) in UCEC using the deseq2 package. The volcano map was drawn by the ggplot2 package with the threshold values of |log2 fold-change (FC)| > 1.0 and adjusted *p*-value < 0.05. Then, we performed GO and KEGG enrichment analyses of DEGs using the ggplot2 package for visualization and the cluster Profiler package for statistical analysis. Furthermore, we built a PPI network of DEGs obtained with the threshold values of |log2 fold-change (FC)| > 2.0 using STRING web and analyzed the hub genes by the MCC algorithm of CytoHubba in Cytoscape (version 3.7.2).

## Results

### CLDN6 Expression in Pan-Cancer

**FIGURE 1:**
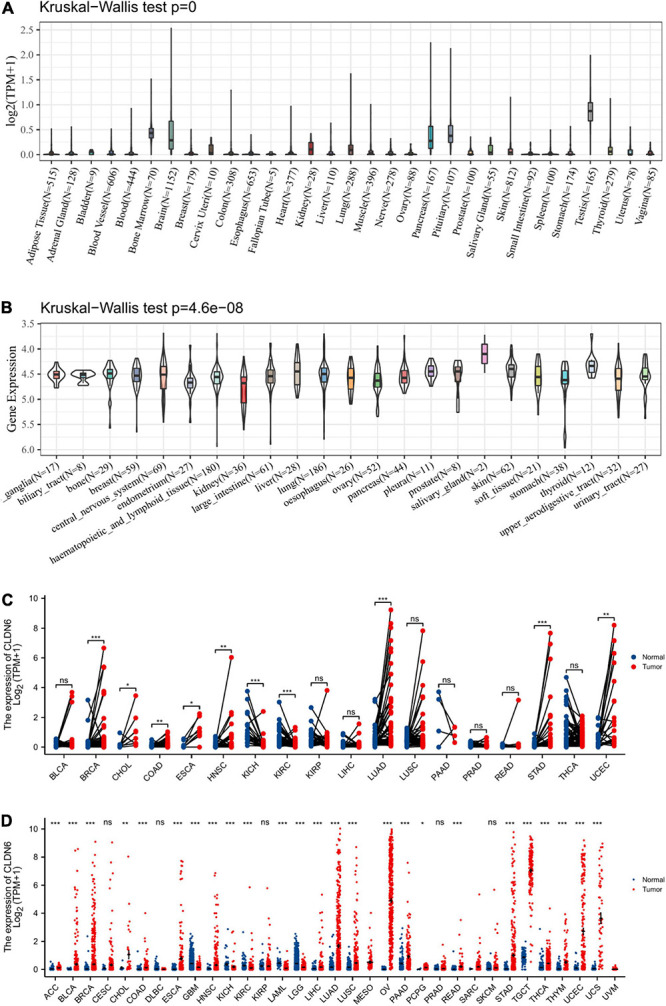
Expression level of CLDN6 gene in tumors and normal tissues. **(A)** CLDN6 expression in normal tissues; **(B)** CLDN6 expression in tumor cell lines; **(C)** CLDN6 expression in TCGA tumors and adjacent normal tissues; **(D)** CLDN6 expression in TCGA tumors and normal tissues with the data of the GTEx database as controls (*p < 0.05, **p < 0.01, ***p < 0.001).

We displayed CLDN6 expression in normal tissues from the GTEx database and found that CLDN6 was less expressed across most normal tissues, and the highest expression tissue was the testis (Figure 1A). In contrast, CLDN6 was expressed more in almost all cell lines (Figure 1B). For TCGA tumors and adjacent normal tissues, CLDN6 expression was significantly upregulated in eight cancer types, including breast invasive carcinoma (BRCA), cholangiocarcinoma (CHOL), colon adenocarcinoma (COAD), esophageal carcinoma (ESCA), head and neck squamous cell carcinoma (HNSC), lung adenocarcinoma (LUAD), STAD, and UCEC, while it was downregulated in kidney chromophobe (KICH) and kidney renal clear cell carcinoma (KIRC) (Figure 1C). Furthermore, for TCGA tumors with the data of the GTEx database as controls, CLDN6 expression was significantly upregulated in 20 cancer types, including ACC, BLCA, BRCA, CHOL, COAD, ESCA, HNSC, liver hepatocellular carcinoma (LIHC), LUAD, lung squamous cell carcinoma (LUSC), OV, pancreatic adenocarcinoma (PAAD), pheochromocytoma and paraganglioma (PCPG), rectum adenocarcinoma (READ), STAD, TGCT, thyroid carcinoma (THCA), thymoma (THYM), UCEC, and UCS, while it was downregulated in glioblastoma multiforme (GBM), KICH, KIRC, LAML, and brain lower grade glioma (LGG) (Figure 1D).

### Correlations Between CLDN6 and Molecular or Immune Subtypes of Cancers

**FIGURE 2:**
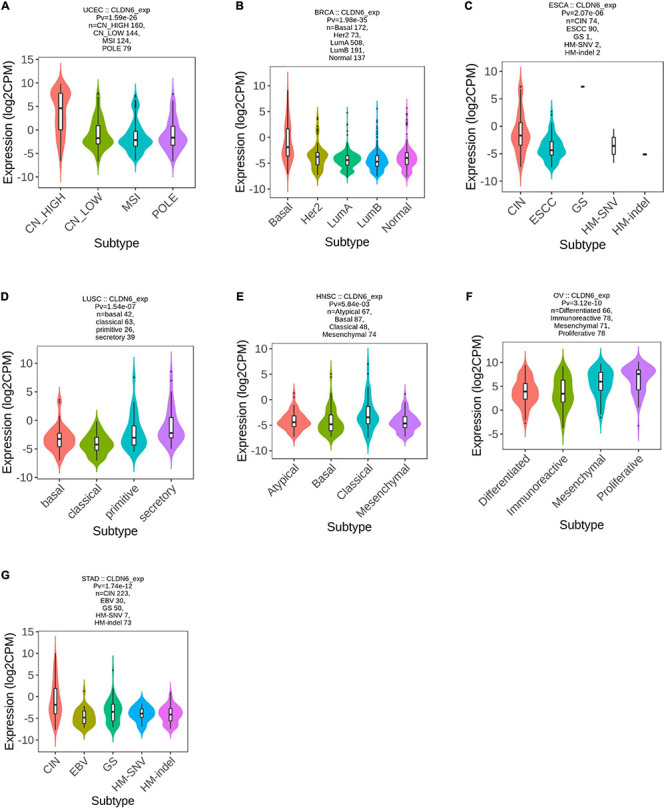
Correlations between CLDN6 expression and molecular subtypes across TCGA tumors. **(A)** UCEC; **(B)** BRCA; **(C)** ESCA; **(D)** LUSC; **(E)** HNSC; **(F)** OV; **(G)** STAD.

We explored the correlations between CLDN6 differential expression and molecular subtypes in pan-cancer from the TISIDB database and found that CLDN6 was expressed differently in different molecular subtypes of seven cancer types, including UCEC, BRCA, ESCA, LUSC, HNSC, OV, and STAD. Moreover, for UCEC, CLDN6 was identified to express more in the molecular subtype of CN_HIGH than other molecular subtypes (Figure 2A). For BRCA, CLDN6 was expressed the highest in the molecular subtype of basal (Figure 2B). For ESCA, CLDN6 was expressed the highest in the molecular subtype of CIN (Figure 2C). For LUSC, CLDN6 was expressed the highest in the molecular subtype of secretory (Figure 2D). For HNSC, CLDN6 was expressed the highest in the molecular subtype of classical (Figure 2E). For OV, CLDN6 was expressed the highest in the molecular subtype of proliferative (Figure 2F). For STAD, CLDN6 was expressed the highest in the molecular subtype of CIN (Figure 2G).

**FIGURE 3:**
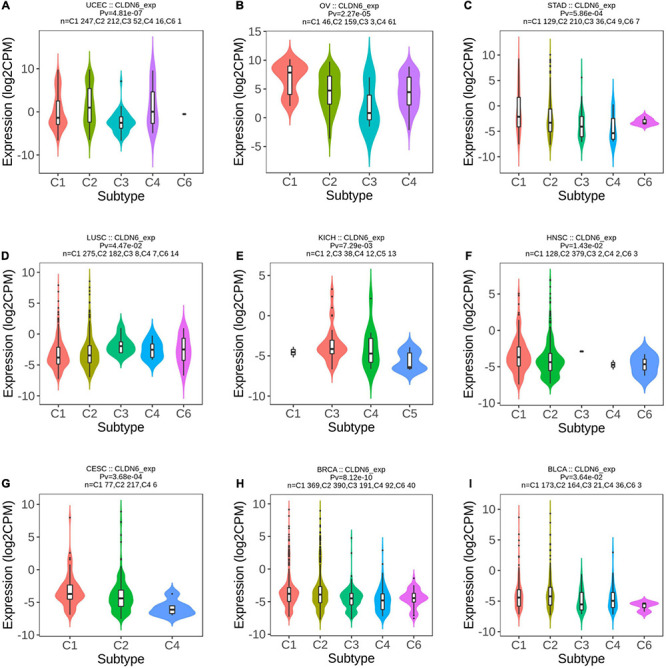
Correlations between CLDN6 expression and immune subtypes across TCGA tumors. **(A)** UCEC; **(B)** OV; **(C)** STAD; **(D)** LUSC; **(E)** KICH; **(F)** HNSC; **(G)** CESC; **(H)** BRCA; **(I)** BLCA.

Meanwhile, we observed that CLDN6 expression was significantly correlated with different immune subtypes (C1: wound healing, C2: IFN-gamma dominant, C3: inflammatory, C4: lymphocyte depleted, C5: immunologically quiet, C6: TGF-b dominant) of nine cancer types, including UCEC (Figure 3A), OV (Figure 3B), STAD (Figure 3C), LUSC (Figure 3D), KICH (Figure 3E), HNSC (Figure 3F), cervical squamous cell carcinoma and endocervical adenocarcinoma (CESC) (Figure 3G), BRCA (Figure 3H), and BLCA (Figure 3I). Also, we observed that CLDN6 expression was associated with immune stimulators (Supplementary Figure 1) and immune inhibitors (Supplementary Figure 2) in the majority of malignant tumors except for KIRC, kidney renal papillary cell carcinoma (KIRP), LGG, LIHC, PCPG, prostate adenocarcinoma (PRAD), READ, sarcoma (SARC), and skin cutaneous melanoma (SKCM).

### PPI Network and GO and KEGG Enrichment Analyses

**FIGURE 4:**
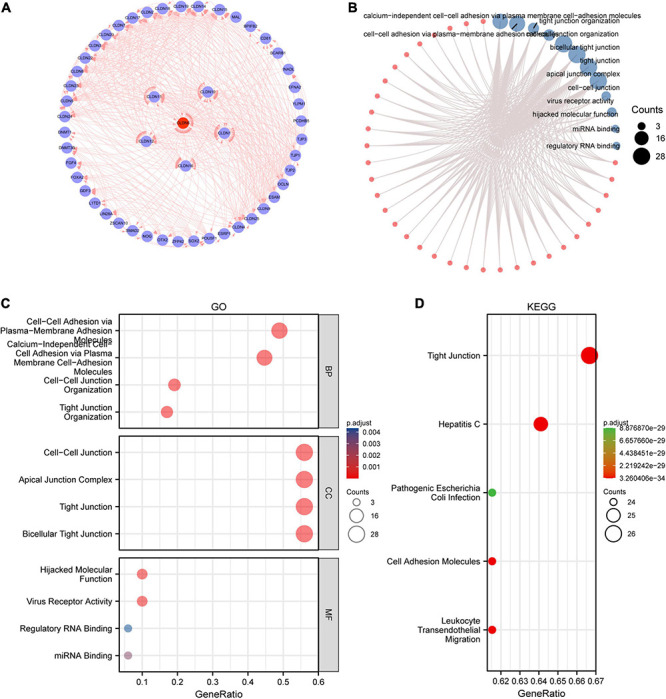
Protein–protein interaction (PPI) network, GO analysis, and KEGG analysis of 50 targeted binding proteins of CLDN6. **(A)** PPI network; **(B)** visual network of GO and KEGG analyses; **(C)** GO analysis; **(D)** KEGG analysis.

We screened out 50 targeted binding proteins of CLDN6 using the STRING database and Cytoscape (Figure 4A). Then, we conducted the GO enrichment analysis (Figure 4B) of 50 targeted binding proteins, revealing that the primary biological process (BP) contained calcium-independent cell–cell adhesion *via* plasma membrane cell adhesion molecules, cell–cell adhesion *via* plasma membrane adhesion molecules, tight junction organization, and cell–cell junction organization. The cellular component (CC) was mainly enriched in bicellular tight junction, apical junction complex, tight junction, and cell–cell junction. The molecular function (MF) was primarily involved in virus receptor activity, hijacked MF, miRNA binding, and regulatory RNA binding. The KEGG pathway enrichment (Figures 4C,D) was mainly related to leukocyte transendothelial migration, tight junction, cell adhesion molecules, hepatitis C, and pathogenic *Escherichia coli* infection.

### Diagnostic Value of CLDN6 in Pan-Cancer

**FIGURE 5:**
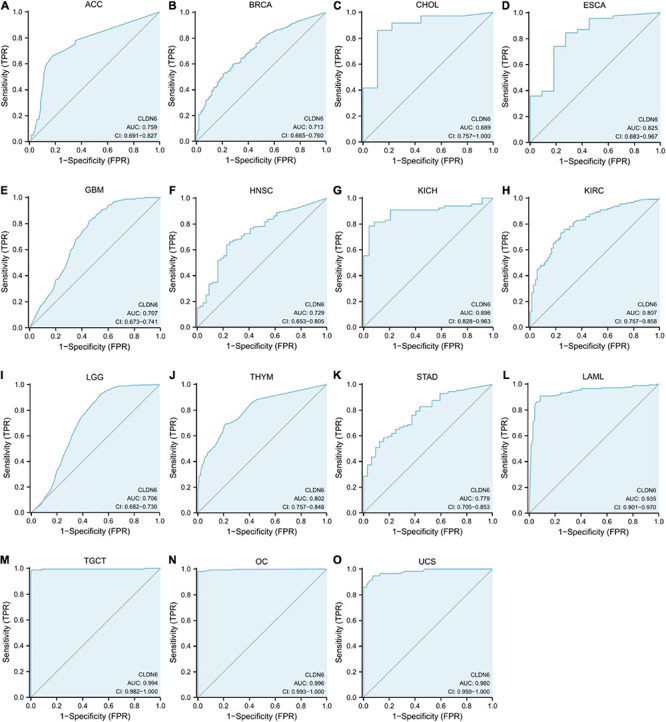
Receiver operating characteristic (ROC) curve for CLDN6 expression in pan-cancer. **(A)** ACC; **(B)** BRCA; **(C)** CHOL; **(D)** ESCA; **(E)** GBM; **(F)** HNSC; **(G)** KICH; **(H)** KIRC; **(I)** LGG; **(J)** THYM; **(K)** STAD; **(L)** LAML; **(M)** TGCT; **(N)** OC; **(O)** UCS.

The ROC curve was performed to assess the diagnostic value of CLDN6 in pan-cancer. The results showed that CLDN6 had a certain accuracy (AUC > 0.7) in predicting 15 cancer types, including ACC (AUC = 0.759) (Figure 5A), BRCA (AUC = 0.713) (Figure 5B), CHOL (AUC = 0.889) (Figure 5C), ESCA (AUC = 0.825) (Figure 5D), GBM (AUC = 0.707) (Figure 5E), HNSC (AUC = 0.729) (Figure 5F), KICH (AUC = 0.896) (Figure 5G), KIRC (AUC = 0.807) (Figure 5H), LGG (AUC = 0.706) (Figure 5I), THYM (AUC = 0.802) (Figure 5J), STAD (AUC = 0.779) (Figure 5K), LAML (AUC = 0.935) (Figure 5L), TGCT (AUC = 0.994) (Figure 5M), OC (AUC = 0.996) (Figure 5N), and UCS (AUC = 0.980) (Figure 5O). Among them, CLDN6 had a high accuracy (AUC > 0.9) in predicting LAML, TGCT, OC, and UCS.

### Prognostic Value of CLDN6 in Cancers

**FIGURE 6:**
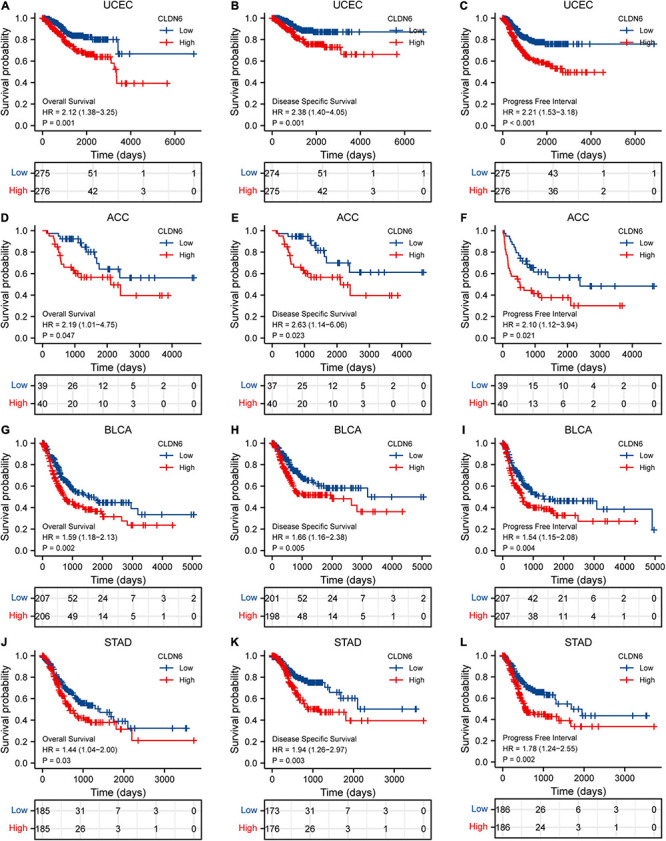
Correlations between CLDN6 expression and the prognosis (OS, DSS, and PFI) of cancers. **(A–C)** UCEC; **(D–F)** ACC; **(G–I)** BLCA; **(J–L)** STAD.

The expression level of CLDN6 was notably correlated with the OS, DSS, and PFI of UCEC, ACC, BLCA, and STAD. For UCEC, Cox regression results showed that the higher expression of CLDN6 had a worse prognosis, including OS [hazard ratio (HR) = 2.12, 95% confidence interval (CI): 1.38–3.25, *p* = 0.001] (Figure 6A), DSS (HR = 2.38, 95% CI: 1.40–4.05, *p* = 0.001) (Figure 6B), and PFI (HR = 2.21, 95% CI: 1.53–3.18, *p* < 0.001) (Figure 6C). Also, for ACC, Cox regression results showed that the higher expression of CLDN6 had a worse prognosis, including OS (HR = 2.19, 95% CI: 1.01–4.75, *p* = 0.047) (Figure 6D), DSS (HR = 2.63, 95% CI: 1.14–6.06, *p* = 0.023) (Figure 6E), and PFI (HR = 2.10, 95% CI: 1.12–3.94, *p* = 0.021) (Figure 6F). For BLCA, Cox regression results showed that the higher expression of CLDN6 had a worse prognosis, including OS (HR = 1.59, 95% CI: 1.18–2.13, *p* = 0.002) (Figure 6G), DSS (HR = 1.66, 95% CI: 1.16–2.38, *p* = 0.005) (Figure 6H), and PFI (HR = 1.54, 95% CI: 1.15–2.08, *p* = 0.004) (Figure 6I). For STAD, Cox regression results showed that the higher expression of CLDN6 had a worse prognosis, including OS (HR = 1.44, 95% CI: 1.04–2.00, *p* = 0.03) (Figure 6J), DSS (HR = 1.94, 95% CI: 1.26–2.97, *p* = 0.003) (Figure 6K), and PFI (HR = 1.78, 95% CI: 1.24–2.55, *p* = 0.002) (Figure 6L).

**FIGURE 7:**
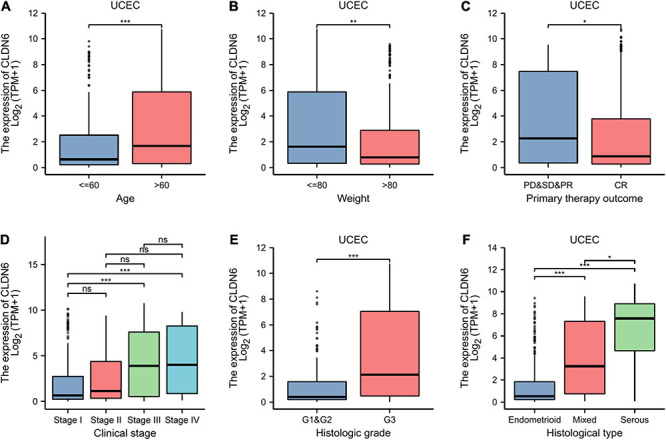
Associations between CLDN6 expression and different clinical characteristics in UCEC. **(A)** Age; **(B)** weight; **(C)** primary therapy outcome; **(D)** clinical stage; **(E)** histologic grade; **(F)** histological type. ns, *p* ≥ 0.05; *p < 0.05; **p < 0.01; ***p < 0.001.

**FIGURE 8:**
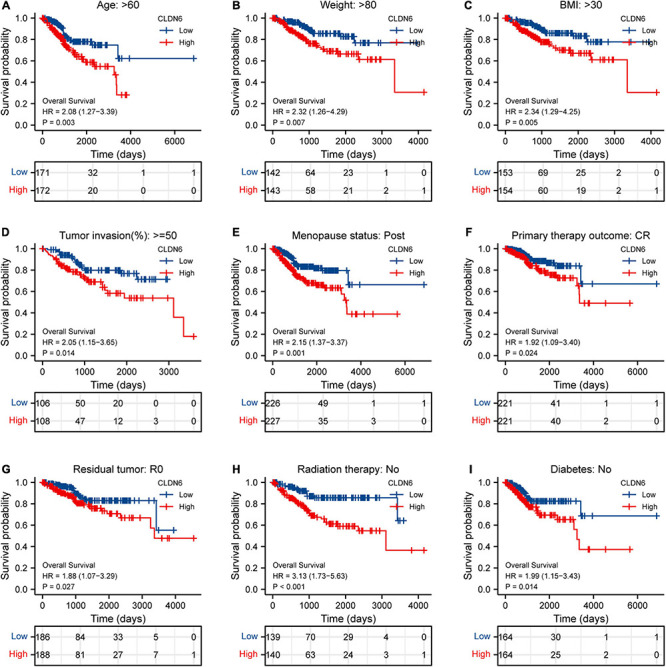
Associations between CLDN6 expression and the OS in different clinical subgroups of UCEC. **(A)** Age > 60; **(B)** weight > 80; **(C)** BMI > 30; **(D)** tumor invasion ≥ 50%; **(E)** postmenopause; **(F)** primary therapy outcome (CR); **(G)** residual tumor (R0); **(H)** radiation therapy (No); **(I)** diabetes (No).

**FIGURE 9:**
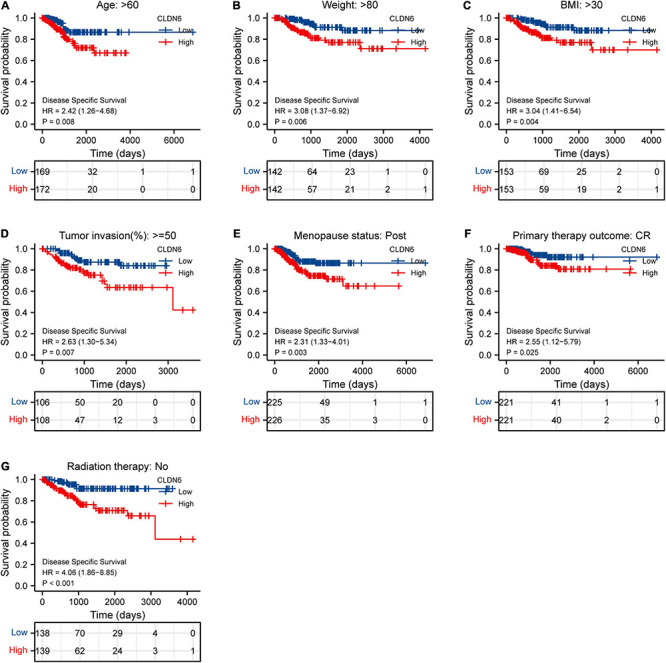
Associations between CLDN6 expression and the DSS in different clinical subgroups of UCEC. **(A)** Age > 60; **(B)** weight > 80; **(C)** BMI > 30; **(D)** tumor invasion ≥ 50%; **(E)** postmenopause; **(F)** primary therapy outcome (CR); **(G)** radiation therapy (No).

Furthermore, we investigated the correlations between CLDN6 and prognosis (OS, DSS, and PFI) in different clinical subgroups of UCEC. The results showed that the higher expression of CLDN6 had a worse OS in most clinical subgroups, including subgroup of age > 60 (Figure 7A), subgroup of weight > 80 (Figure 7B), subgroup of BMI > 30 (Figure 7C), subgroup of tumor invasion ≥ 50% (Figure 7D), subgroup of postmenopause (Figure 7E), subgroup of primary therapy outcome (CR) (Figure 7F), subgroup of residual tumor (R0) (Figure 7G), subgroup of radiation therapy (No) (Figure 7H), and subgroup of diabetes (No) (Figure 7I). For DSS, the higher expression of CLDN6 had a worse DSS in subgroup of age > 60 (Figure 8A), subgroup of weight > 80 (Figure 8B), subgroup of BMI > 30 (Figure 8C), subgroup of tumor invasion ≥ 50% (Figure 8D), subgroup of postmenopause (Figure 8E), subgroup of primary therapy outcome (CR) (Figure 8F), and subgroup of radiation therapy (No) (Figure 8G). For PFI, the higher expression of CLDN6 had a worse PFI in subgroup of weight > 80 (Figure 9A), subgroup of BMI > 30 (Figure 9B), subgroup of postmenopause (Figure 9C), subgroup of primary therapy outcome (CR) (Figure 9D), subgroup of residual tumor (R0) (Figure 9E), and subgroup of clinical stage III (Figure 9F).

### CLDN6 Is Correlated With Different Clinical Characteristics in UCEC

**FIGURE 10:**
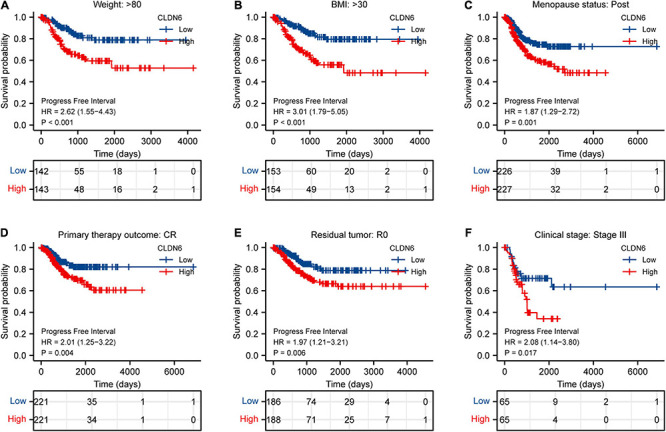
Associations between CLDN6 expression and the PFI in different clinical subgroups of UCEC. **(A)** Weight > 80; **(B)** BMI > 30; **(C)** postmenopause; **(D)** primary therapy outcome (CR); **(E)** residual tumor (R0); **(F)** stage III.

We further presented the associations between CLDN6 and different clinical characteristics in UCEC and found that CLDN6 expression was significantly related to age, stage, weight, histological type, histologic grade, and menopause status of UCEC (Table 1). Moreover, CLDN6 was expressed higher in patients with age > 60 (Figure 10A), stage III/IV (Figure 10D), histologic grade 3 (Figure 10E), and histological type of serous (Figure 10F), while it was expressed lower in patients with weight > 80 (Figure 10B) and primary therapy outcome (CR) (Figure 10C), respectively.

**TABLE 1:** Clinical characteristics of UCEC patients.

**Characteristic**	**Low expression of CLDN6**	**High expression of CLDN6**	***p***
***n***	**276**	**276**	
Clinical stage, n (%)			<0.001
Stage I	196 (35.5%)	146 (26.4%)	
Stage II	25 (4.5%)	26 (4.7%)	
Stage III	47 (8.5%)	83 (15%)	
Stage IV	8 (1.4%)	21 (3.8%)	
Age, *n* (%)			<0.001
≤60	124 (22.6%)	82 (14.9%)	
>60	150 (27.3%)	193 (35.2%)	
Weight, n (%)			0.036
≤80	109 (20.6%)	134 (25.4%)	
>80	155 (29.4%)	130 (24.6%)	
Histological type, *n* (%)			<0.001
Endometrioid	257 (46.6%)	153 (27.7%)	
Mixed	8 (1.4%)	16 (2.9%)	
Serous	11 (2%)	107 (19.4%)	
Histologic grade, *n* (%)			<0.001
G1	68 (12.6%)	30 (5.5%)	
G2	83 (15.3%)	37 (6.8%)	
G3	122 (22.6%)	201 (37.2%)	
Menopause status, *n* (%)			0.005
Pre	17 (3.4%)	18 (3.6%)	
Peri	15 (3%)	2 (0.4%)	
Post	217 (42.9%)	237 (46.8%)	

### Univariate and Multivariate Cox Regression Analyses in UCEC

We implemented univariate and multivariate Cox regression analyses of CLDN6 and clinical characteristics in UCEC. In the univariate and multivariate Cox regression analyses, clinical stage, primary therapy outcome, age, and CLDN6 were significantly associated with the OS (Table 2), while clinical stage, primary therapy outcome, histologic grade, and CLDN6 were significantly associated with DSS (Supplementary Table 1), and clinical stage, primary therapy outcome, and CLDN6 were significantly associated with PFI (Supplementary Table 2).

**TABLE 2:** Univariate and multivariate Cox regression analyses of clinical characteristics associated with OS of UCEC.

**Characteristics**	**Total (*N*)**	**Univariate analysis**		**Multivariate analysis**	
		**Hazard ratio (95% CI)**	***p*-Value**	**Hazard ratio (95% CI)**	***p*-Value**
Clinical stage (stage II and stage III and stage IV vs. stage I)	551	3.270 (2.145–4.984)	<0.001	3.225 (1.940–5.362)	<0.001
Primary therapy outcome (CR vs. PD and SD and PR)	480	0.129 (0.078–0.215)	<0.001	0.168 (0.100–0.283)	<0.001
Age (>60 vs. ≤60)	549	1.847 (1.160–2.940)	0.010	1.754 (1.049–2.931)	0.032
CLDN6 (high vs. low)	551	2.121 (1.383–3.250)	<0.001	1.669 (1.020–2.732)	0.041

### Co-expression Gene Analysis of CLDN6 in UCEC

**FIGURE 11:**
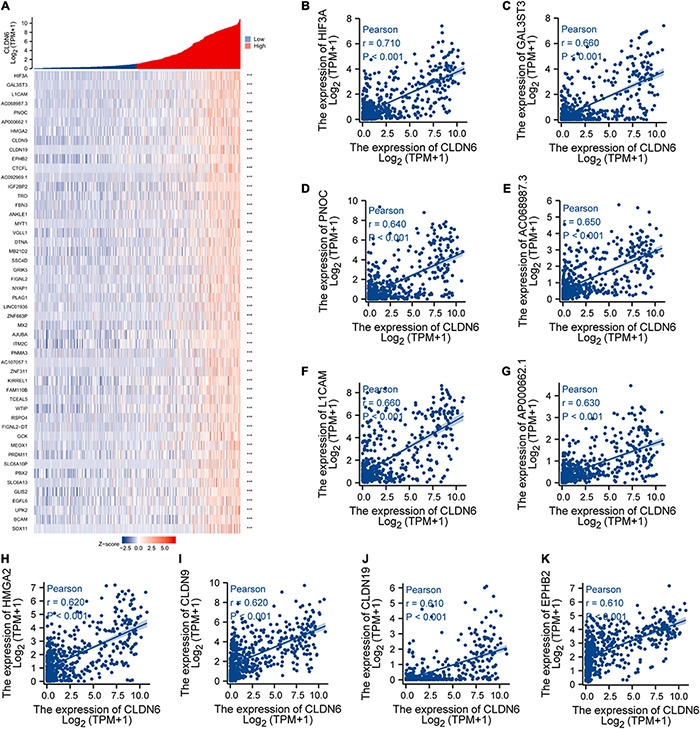
Top 50 genes positively correlated with CLDN6 expression in UCEC. **(A)** The gene co-expression heatmap of the top 50 genes positively correlated with CLDN6 in UCEC; **(B–K)** correlation analysis of the top 10 genes and CLDN6 in the heatmap.

**FIGURE 12:**
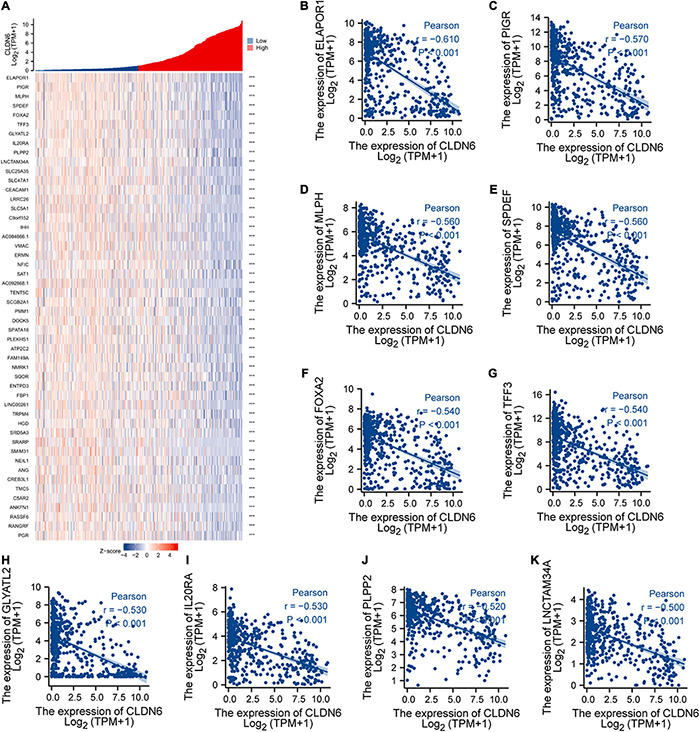
Top 50 genes negatively correlated with CLDN6 expression in UCEC. **(A)** The gene co-expression heatmap of the top 50 genes negatively correlated with CLDN6 in UCEC; **(B–K)** correlation analysis of the top 10 genes and CLDN6 in the heatmap.

We explored the top 50 co-expression genes positively or negatively correlated with CLDN6 expression in UCEC and displayed the correlations between CLDN6 expression and top 10 genes expression in the heatmap. In the heatmap of positive correlation (Figure 11A), we obtained the top 10 genes, including HIF3A (*r* = 0.71) (Figure 11B), GAL3ST3 (*r* = 0.66) (Figure 11C), PNOC (*r* = 0.64) (Figure 11D), AC068987.3 (*r* = 0.65) (Figure 11E), L1CAM (*r* = 0.66) (Figure 11F), AP000662.1 (*r* = 0.63) (Figure 11G), HMGA2 (*r* = 0.62) (Figure 11H), CLDN9 (*r* = 0.62) (Figure 11I), CLDN19 (*r* = 0.61) (Figure 11J), and EPHB2 (*r* = 0.61) (Figure 11K). In the heatmap of negative correlation (Figure 12A), we obtained the top 10 genes, including ELAPOR1 (*r* = −0.61) (Figure 12B), PIGR (*r* = −0.57) (Figure 12C), MLPH (*r* = −0.56) (Figure 12D), SPDEF (*r* = −0.56) (Figure 12E), FOXA2 (*r* = −0.54) (Figure 12F), TFF3 (*r* = −0.54) (Figure 12G), GLYATL2 (*r* = −0.53) (Figure 12H), IL20RA (*r* = −0.53) (Figure 12I), PLPP2 (*r* = −0.52) (Figure 12J), and LNCTAM34A (*r* = −0.50) (Figure 12K).

### DEGs Between CLDN6 High Expression Group and Low Expression Groups in UCEC

**FIGURE 13:**
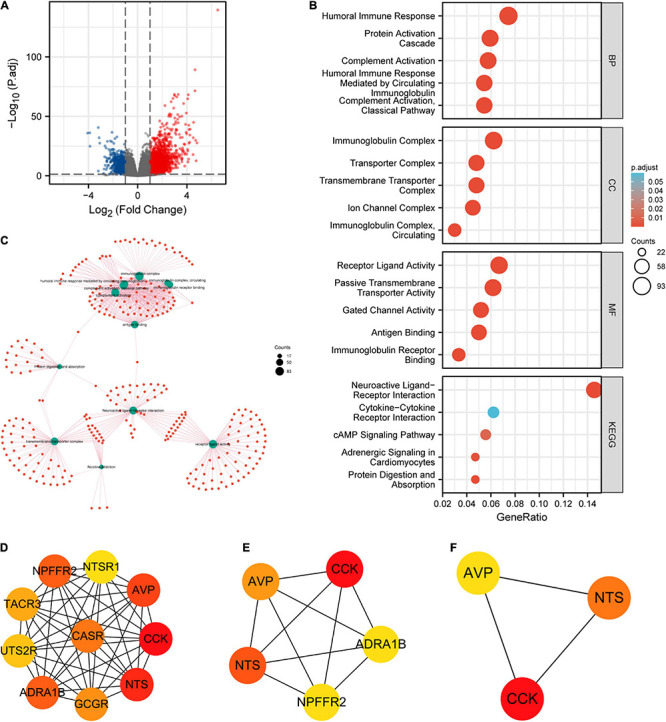
Protein–protein interaction (PPI) network building and GO and KEGG analyses of DEGs between CLDN6 high expression and low expression groups in UCEC. **(A)** The volcano map of DEGs (red: upregulation; blue: downregulation); **(B,C)** GO and KEGG analyses of DEGs; **(D–F)** hub genes of PPI network and MCODE2 components identified in the gene lists.

A total of 2,884 DEGs were acquired with the threshold values of |log2 fold-change (FC)| > 1.0 and adjusted *p*-value < 0.05, including 2,005 upregulated genes and 879 downregulated genes (Figure 13A). Among them, 446 DEGs were obtained with the threshold values of |log2 fold-change (FC)| > 2.0 and adjusted *p*-value < 0.05, including 388 upregulated genes and 58 downregulated genes. Then, we conducted the GO and KEGG enrichment analyses (Figures 13B,C) of DEGs, revealing that the primary BP contained complement activation, classical pathway, humoral immune response mediated by circulating immunoglobulin, complement activation, protein activation cascade, and humoral immune response. The CC was mainly enriched in immunoglobulin complex, immunoglobulin complex, circulating, transmembrane transporter complex, transporter complex, and ion channel complex. The MF was primarily involved in antigen binding, immunoglobulin receptor binding, receptor ligand activity, gated channel activity, and passive transmembrane transporter activity. The KEGG pathway enrichment was mainly related to neuroactive ligand–receptor interaction, cytokine–cytokine receptor interaction, cAMP signaling pathway, adrenergic signaling in cardiomyocytes, and protein digestion and absorption.

Furthermore, we obtained the top 10 hub genes (Figure 13D) of 446 DEGs, including CCK, NTS, AVP, ADRA1B, NPFFR2, CASR, GCGR, TACR3, UTS2R, and NTSR1. Among them, the top five hub genes (Figure 13E) were CCK, NTS, AVP, ADRA1B, and NPFFR2. Additionally, the top three hub genes (Figure 13F) were CCK, NTS, and AVP.

## Discussion

Claudins, together with occludins and junctional adhesion molecules, are three transmembrane components of tight junctions, playing the role of paracellular barrier and intracellular signaling, while regulating the proliferation, differentiation, and apoptosis of the epithelial cell (Severson and Parkos, 2009; Liu F. et al., 2016; Osanai et al., 2017; Kage et al., 2019; Phattarataratip and Sappayatosok, 2020). It has been demonstrated that claudins are intimately involved in the majority of epithelial-derived cancers by altering their expression manners (Baumholtz et al., 2017; Osanai et al., 2017; Singh et al., 2017; Tabaries and Siegel, 2017; Cherradi et al., 2019; Gowrikumar et al., 2019; Kage et al., 2019; Auguste et al., 2020; Koziel et al., 2020; Phattarataratip and Sappayatosok, 2020; Kolchakova et al., 2021). Recent studies have confirmed that CLDN6 could promote the cell migration and invasion of certain cancers, including breast cancer (Liu Y. et al., 2016; Lu et al., 2017), gastric cancer (Torres-Martinez et al., 2017; Yu et al., 2019), hepatocellular carcinoma (Huang et al., 2020; Kong et al., 2020), and endometrial cancer (Kojima et al., 2021), and can be vigorously stimulated in human-induced pluripotent stem cells developed from fibroblasts, thus might present to be a promising target candidate antibody for cancer therapy (Türeci et al., 2018).

However, there is no existing study to our knowledge to evaluate the significance of CLDN6 in pan-cancer on the whole scale. In the present study, to assess the expression level of CLDN6 across pan-cancer, we examined CCLE database, TCGA database, and GTEx database and found that it was significantly upregulated in 20 types of human cancers, including ACC, BLCA, BRCA, CHOL, COAD, ESCA, HNSC, LIHC, LUAD, LUSC, OV, PAAD, PCPG, READ, STAD, TGCT, THCA, THYM, UCEC, and UCS, while it was downregulated in GBM, KICH, KIRC, LAML, and LGG. The finding reveals that CLDN6 serves as a cancer-promoting gene in the majority of malignant tumors and might be involved in tumor formation or cancer development. In addition, there are meaningful associations between CLDN6 expression level and molecular subtypes of seven cancer types, including UCEC, BRCA, ESCA, LUSC, HNSC, OV, and STAD. For instance, CLDN6 was observed to express the highest in the molecular subtype of CN_HIGH in UCEC, in the molecular subtype of basal in BRCA, and in the molecular subtype of CIN in STAD. Meanwhile, we observed that CLDN6 was significantly correlated with different immune subtypes in nine cancer types. It is worth pointing out that CLDN6 is closely correlated with both molecular subtype and immune subtype in six types of cancers, including UCEC, BRCA, STAD, OV, LUSC, and HNSC. As the previous study demonstrated, CLDN6 is confirmed to be a possible single prognostic marker and promising therapeutic target for a subgroup of intestinal type gastric cancer (Kohmoto et al., 2020). Hence, the research focused on a distinct molecular subtype or immune subtype of cancers may provide an appropriate entry point to explore the role of CLDN6. Then, to examine the diagnostic and prognostic value of CLDN6, we performed the ROC curve and the Kaplan–Meier survival curve in pan-cancer and found that CLDN6 had a certain accuracy (AUC > 0.7) in predicting 15 cancer types, especially had a high accuracy (AUC > 0.9) in predicting LAML, TGCT, OV, and UCS. Additionally, CLDN6 was significantly correlated with the OS, DSS, and PFI in UCEC, ACC, BLCA, and STAD. The results demonstrate here that CLDN6 presents great diagnostic and prognostic significance in the above cancers and may be a potential biomarker or therapeutic target for precision oncology. To deeply investigate the biofunction of CLDN6, we conducted the GO and KEGG pathway enrichment analyses of its 50 targeted binding proteins, revealing that the BP was major in cell–cell adhesion or bicellular tight junction, its MF was primarily involved in virus receptor activity, hijacked MF, miRNA binding, and regulatory RNA binding, and the main pathways were enriched in leukocyte transendothelial migration, tight junction, cell adhesion molecules, hepatitis C, and pathogenic *E. coli* infection. It should be emphasized that CLDN6 is not only essential for cell–cell adhesion and tight junction but also crucial for outside–in cell signaling.

Furthermore, we primarily analyzed the role of CLDN6 played in UCEC and identified the significant correlations between CLDN6 expression level and age, stage, weight, histological type, histologic grade, and menopause status. Subsequently, we discovered that CLDN6 high expression could cause a worse OS, DSS, or PFI in a variety of clinical subgroups of UCEC, yet cause a worse all of the OS, DSS, and PFI only in clinical subgroups of weight > 80, BMI > 30, postmenopause, or primary therapy outcome of CR. Since then, we confirmed clinical stage, primary therapy outcome, and CLDN6 expression level as independent risk factors in OS, DSS, and PFI of UCEC through univariate and multivariate Cox regression analyses. The results are further supported by the prior research involving prognostic significance of aberrant CLDN6 expression in UCEC (Kojima et al., 2020), which established a monoclonal antibody, then performed immunohistochemical staining and semi-quantification to assess the associations between CLDN6 and clinical characteristics in 173 cases, and finally identified stages III/IV, distant metastasis, and high CLDN6 expression as independent prognostic variables for the OS. It is of great importance that our findings provide a more comprehensive and further supplement and strengthen the role of CLDN6 played in UCEC, and that we precisely analyze the correlations between CLDN6 expression and different prognosis conditions (OS, DSS, or PFI) in various clinical subgroups of UCEC. In addition, we further obtained co-expression genes of CLDN6, and the top 10 co-expression genes positively correlated with CLDN6 contained HIF3A, GAL3ST3, PNOC, AC068987.3 (Lnc-SCN8A-2), L1CAM, AP000662.1 (Lnc-CLP1-3), HMGA2, CLDN9, CLDN19, and EPHB2, whereas the top 10 co-expression genes negatively correlated with CLDN6 contained ELAPOR1, PIGR, MLPH, SPDEF, FOXA2, TFF3, GLYATL2, IL20RA, PLPP2, and LNCTAM34A. Besides, we explored the DEGs between CLDN6 high expression group and low expression group, then performed the GO and KEGG pathway analyses of the DEGs, and found that the BP mainly linked to complement activation, classical pathway, humoral immune response mediated by circulating immunoglobulin, complement activation, protein activation cascade, and humoral immune response. The MF was primarily involved in antigen binding, immunoglobulin receptor binding, receptor ligand activity, gated channel activity, and passive transmembrane transporter activity. Finally, we screened out the hub genes of DEGs, including CCK, NTS, AVP, ADRA1B, NPFFR2, CASR, GCGR, TACR3, UTS2R, and NTSR1.

There are still several limitations in the present study. On the one hand, we set out to explore CLDN6 only using CCLE database, TCGA database, and GTEx database while lacking actual clinical data. On the other hand, the precise verification and high-quality evidence should be further performed and provided by biological experiments.

Many computational methods have been applied in bioinformatics research, such as lncRNA–miRNA interaction predictions (Liu et al., 2020; Zhang et al., 2021a, b), the identification of microRNA combinatorial biomarkers (Yang et al., 2017a, b; Liu and Yang, 2018), and so on (Wang et al., 2020; Zhang et al., 2020). In the next step, we will consider the use of the machine learning and deep learning methods in the research of CLDN6. In summary, the identification of CLDN6 in diagnostic and prognostic significance across pan-cancer, together with its further exploration in UCEC, could add a new dimension to the comprehensive understanding of its critical role in tumor promotion and suppression and provide an integrative analyzing basis for deep verification of molecular biology experiments, even for future clinical application of cancer therapies.

## Data Availability Statement

The original contributions presented in the study are included in the article/Supplementary Material, further inquiries can be directed to the corresponding author/s.

## Author Contributions

QZ and LO contributed to the design of the study protocol. CZ performed the statistical analysis and drew the pictures. YL and KL contributed to the writing of the study protocol. CG downloaded the data. All authors approved the final version of the manuscript.

## Conflict of Interest

The authors declare that the research was conducted in the absence of any commercial or financial relationships that could be construed as a potential conflict of interest.

## Publisher’s Note

All claims expressed in this article are solely those of the authors and do not necessarily represent those of their affiliated organizations, or those of the publisher, the editors and the reviewers. Any product that may be evaluated in this article, or claim that may be made by its manufacturer, is not guaranteed or endorsed by the publisher.

## Footnotes

1
https://xenabrowser.net/datapages/

2
https://portals.broadinstitute.org/ccle/

3
https://string-db.org/

## Abbreviations

TCGA: The Cancer Genome Atlas
GTEx: Genotype-Tissue Expression
CCLE: Cancer Cell Line Encyclopedia
DEGs: differentially expressed genes
PPI: protein–protein interaction
GO: Gene Ontology
KEGG: Kyoto Encyclopedia of Genes and Genomes
ROC: receiver operating characteristic
BP: biological process
MF: molecular function
CC: cellular component
AUC: area under the curve
HR: hazard ratio
CI: confidence interval
OS: overall survival
DSS: disease-specific survival
PFI: progression-free interval
TPM: transcripts per million reads
FPKM: fragments per kilobase per million
FC: fold-change
ACC: adrenocortical carcinoma
BLCA: bladder urothelial carcinoma
BRCA: breast invasive carcinoma
CESC: cervical squamous cell carcinoma and endocervical adenocarcinoma
CHOL: cholangiocarcinoma
COAD: colon adenocarcinoma
DLBC: lymphoid neoplasm diffuse large B-cell lymphoma
ESCA: esophageal carcinoma
GBM: glioblastoma multiforme
HNSC: head and neck squamous cell carcinoma
KICH: kidney chromophobe
KIRC: kidney renal clear cell carcinoma
KIRP: kidney renal papillary cell carcinoma
LAML: acute myeloid leukemia
LGG: brain lower grade glioma
LIHC: liver hepatocellular carcinoma
LUAD: lung adenocarcinoma
LUSC: lung squamous cell carcinoma
MESO: mesothelioma
OV: ovarian serous cystadenocarcinoma
PAAD: pancreatic adenocarcinoma
PCPG: pheochromocytoma and paraganglioma
PRAD: prostate adenocarcinoma
READ: rectum adenocarcinoma
SARC: sarcoma
SKCM: skin cutaneous melanoma
STAD: stomach adenocarcinoma
TGCT: testicular germ cell tumors
THCA: thyroid carcinoma
THYM: thymoma
UCEC: uterine corpus endometrial carcinoma
UCS: uterine carcinosarcoma
UVM: uveal melanoma.
