# Supplementary material for: Identification of Claudin-6 as a Molecular Biomarker in Pan-Cancer Through Multiple Omics Integrative Analysis
Source: Front Cell Dev Biol. 2021 Aug 2;9:726656. doi: 10.3389/fcell.2021.726656 (PMC8365468; doi:10.3389/fcell.2021.726656)
Supplement: Supplementary file 1 [file Table_1.DOCX]

| Characteristics | Total(N) | Univariate analysis | |  | Multivariate analysis | |
| --- | --- | --- | --- | --- | --- | --- |
|  |  | Hazard ratio (95% CI) | P value |  | Hazard ratio (95% CI) | P value |
| Clinical stage (Stage II&Stage III&Stage IV vs. Stage I) | 549 | 6.034 (3.372-10.797) | **<0.001** |  | 5.315 (2.412-11.712) | **<0.001** |
| Primary therapy outcome (CR vs. PD&SD&PR) | 480 | 0.074 (0.042-0.129) | **<0.001** |  | 0.105 (0.057-0.192) | **<0.001** |
| Histologic grade (G3 vs. G1&G2) | 538 | 7.851 (3.137-19.651) | **<0.001** |  | 3.154 (1.225-8.124) | **0.017** |
| CLDN6 (High vs. Low) | 549 | 2.384 (1.402-4.053) | **0.001** |  | 1.920 (1.010-3.650) | **0.047** |

**Table S1.** Univariate and multivariate Cox regression analyses of clinical characteristics associated with DSS of UCEC.
