# Supplementary material for: Identification of Claudin-6 as a Molecular Biomarker in Pan-Cancer Through Multiple Omics Integrative Analysis
Source: Front Cell Dev Biol. 2021 Aug 2;9:726656. doi: 10.3389/fcell.2021.726656 (PMC8365468; doi:10.3389/fcell.2021.726656)
Supplement: Supplementary file 2 [file Table_2.DOCX]

| Characteristics | Total(N) | Univariate analysis | |  | Multivariate analysis | |
| --- | --- | --- | --- | --- | --- | --- |
|  |  | Hazard ratio (95% CI) | P value |  | Hazard ratio (95% CI) | P value |
| Clinical stage (Stage II&Stage III&Stage IV vs. Stage I) | 551 | 2.527 (1.780-3.587) | **<0.001** |  | 2.119 (1.406-3.192) | **<0.001** |
| Primary therapy outcome (CR vs. PD&SD&PR) | 480 | 0.120 (0.078-0.184) | **<0.001** |  | 0.147 (0.094-0.231) | **<0.001** |
| Histologic grade (G3 vs. G1&G2) | 540 | 2.088 (1.391-3.136) | **<0.001** |  | 1.139 (0.721-1.802) | 0.577 |
| CLDN6 (High vs. Low) | 551 | 2.209 (1.535-3.180) | **<0.001** |  | 1.878 (1.231-2.864) | **0.003** |

**Table S2.** Univariate and multivariate Cox regression analyses of clinical characteristics associated with PFI of UCEC.
