# Supplementary material for: Identification of Claudin-6 as a Molecular Biomarker in Pan-Cancer Through Multiple Omics Integrative Analysis
Source: Front Cell Dev Biol. 2021 Aug 2;9:726656. doi: 10.3389/fcell.2021.726656 (PMC8365468; doi:10.3389/fcell.2021.726656)
Supplement: Supplementary file 5 [file Table_5.DOCX]

| ONTOLOGY | ID | Description | GeneRatio | BgRatio | pvalue | p.adjust | qvalue |
| --- | --- | --- | --- | --- | --- | --- | --- |
| BP | GO:0016338 | calcium-independent cell-cell adhesion via plasma membrane cell-adhesion molecules | 21/47 | 23/18670 | 3.31e-55 | 3.56e-52 | 2.66e-52 |
| BP | GO:0098742 | cell-cell adhesion via plasma-membrane adhesion molecules | 23/47 | 273/18670 | 2.88e-30 | 1.55e-27 | 1.16e-27 |
| BP | GO:0120193 | tight junction organization | 8/47 | 60/18670 | 2.00e-12 | 7.15e-10 | 5.34e-10 |
| BP | GO:0045216 | cell-cell junction organization | 9/47 | 156/18670 | 1.64e-10 | 4.39e-08 | 3.28e-08 |
| BP | GO:0043297 | apical junction assembly | 7/47 | 64/18670 | 2.24e-10 | 4.81e-08 | 3.59e-08 |
| CC | GO:0005923 | bicellular tight junction | 28/50 | 123/19717 | 5.33e-50 | 3.62e-48 | 3.09e-48 |
| CC | GO:0070160 | tight junction | 28/50 | 128/19717 | 1.86e-49 | 6.33e-48 | 5.39e-48 |
| CC | GO:0043296 | apical junction complex | 28/50 | 143/19717 | 5.84e-48 | 1.32e-46 | 1.13e-46 |
| CC | GO:0005911 | cell-cell junction | 28/50 | 459/19717 | 4.60e-33 | 7.82e-32 | 6.65e-32 |
| CC | GO:0016327 | apicolateral plasma membrane | 6/50 | 18/19717 | 3.54e-12 | 4.81e-11 | 4.09e-11 |
| MF | GO:0001618 | virus receptor activity | 5/50 | 74/17697 | 2.04e-06 | 9.79e-05 | 7.84e-05 |
| MF | GO:0104005 | hijacked molecular function | 5/50 | 74/17697 | 2.04e-06 | 9.79e-05 | 7.84e-05 |
| MF | GO:0035198 | miRNA binding | 3/50 | 31/17697 | 9.02e-05 | 0.003 | 0.002 |
| MF | GO:0061980 | regulatory RNA binding | 3/50 | 39/17697 | 1.81e-04 | 0.004 | 0.003 |
| MF | GO:0031369 | translation initiation factor binding | 2/50 | 32/17697 | 0.004 | 0.071 | 0.056 |

The GO analysis of 50 targeted binding proteins of CLDN6
