# Supplementary material for: Identification of Claudin-6 as a Molecular Biomarker in Pan-Cancer Through Multiple Omics Integrative Analysis
Source: Front Cell Dev Biol. 2021 Aug 2;9:726656. doi: 10.3389/fcell.2021.726656 (PMC8365468; doi:10.3389/fcell.2021.726656)
Supplement: Supplementary file 7 [file Table_7.DOCX]

| ONTOLOGY | ID | Description | GeneRatio | BgRatio | pvalue | p.adjust | qvalue |
| --- | --- | --- | --- | --- | --- | --- | --- |
| KEGG | hsa04670 | Leukocyte transendothelial migration | 24/39 | 114/8076 | 6.39e-36 | 3.26e-34 | 2.89e-34 |
| KEGG | hsa04530 | Tight junction | 26/39 | 169/8076 | 1.94e-35 | 4.94e-34 | 4.38e-34 |
| KEGG | hsa05160 | Hepatitis C | 25/39 | 157/8076 | 2.75e-34 | 4.67e-33 | 4.14e-33 |
| KEGG | hsa04514 | Cell adhesion molecules | 24/39 | 149/8076 | 7.09e-33 | 9.04e-32 | 8.02e-32 |
| KEGG | hsa05130 | Pathogenic Escherichia coli infection | 24/39 | 197/8076 | 8.70e-30 | 8.88e-29 | 7.88e-29 |

The KEGG analysis of 50 targeted binding proteins of CLDN6
