# Supplementary figures and images for: Identification of Claudin-6 as a Molecular Biomarker in Pan-Cancer Through Multiple Omics Integrative Analysis
Source: Front Cell Dev Biol. 2021 Aug 2;9:726656. doi: 10.3389/fcell.2021.726656 (PMC8365468; doi:10.3389/fcell.2021.726656)

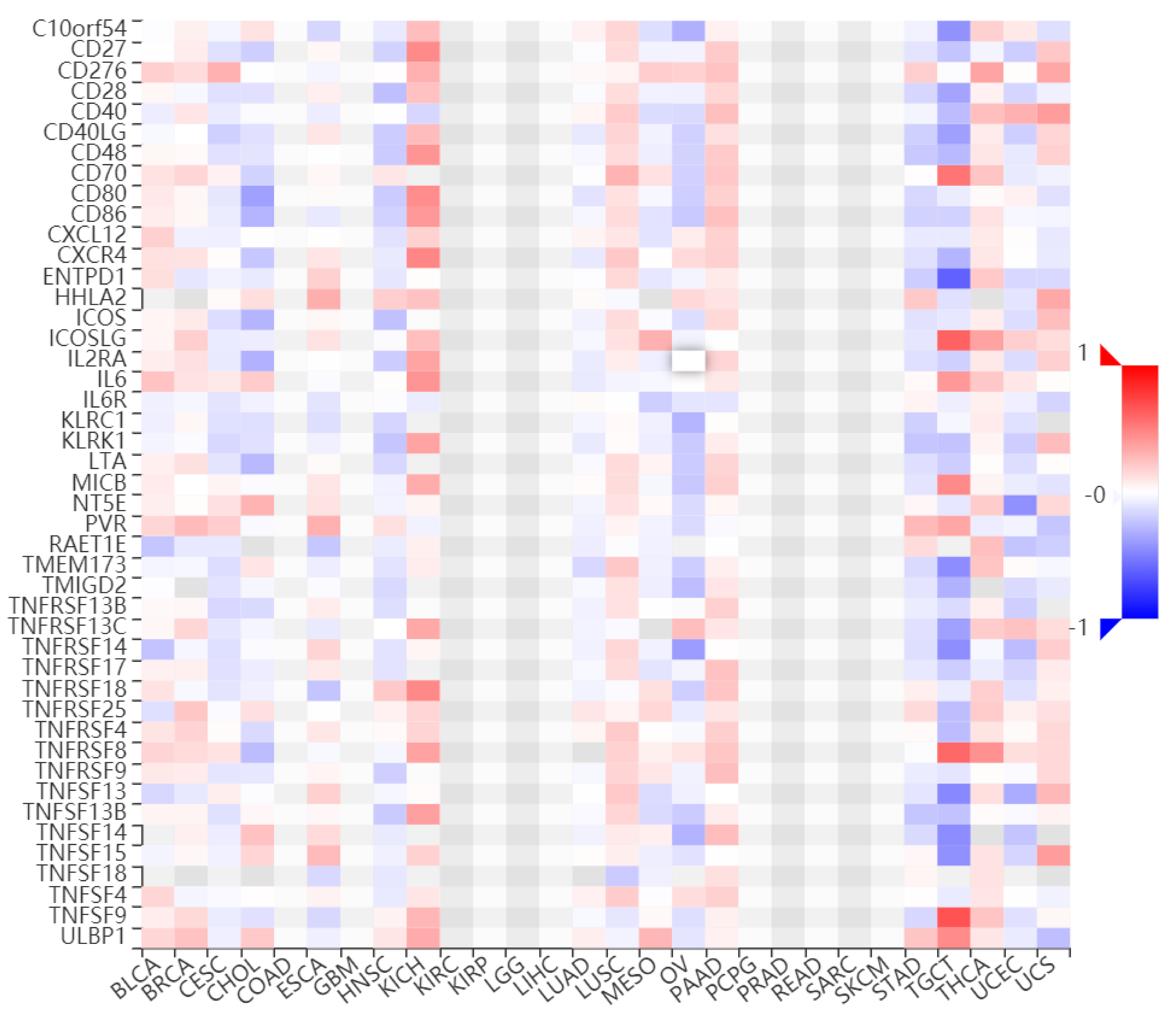

Supplement: Supplementary file 13 [file Image_1.TIF]

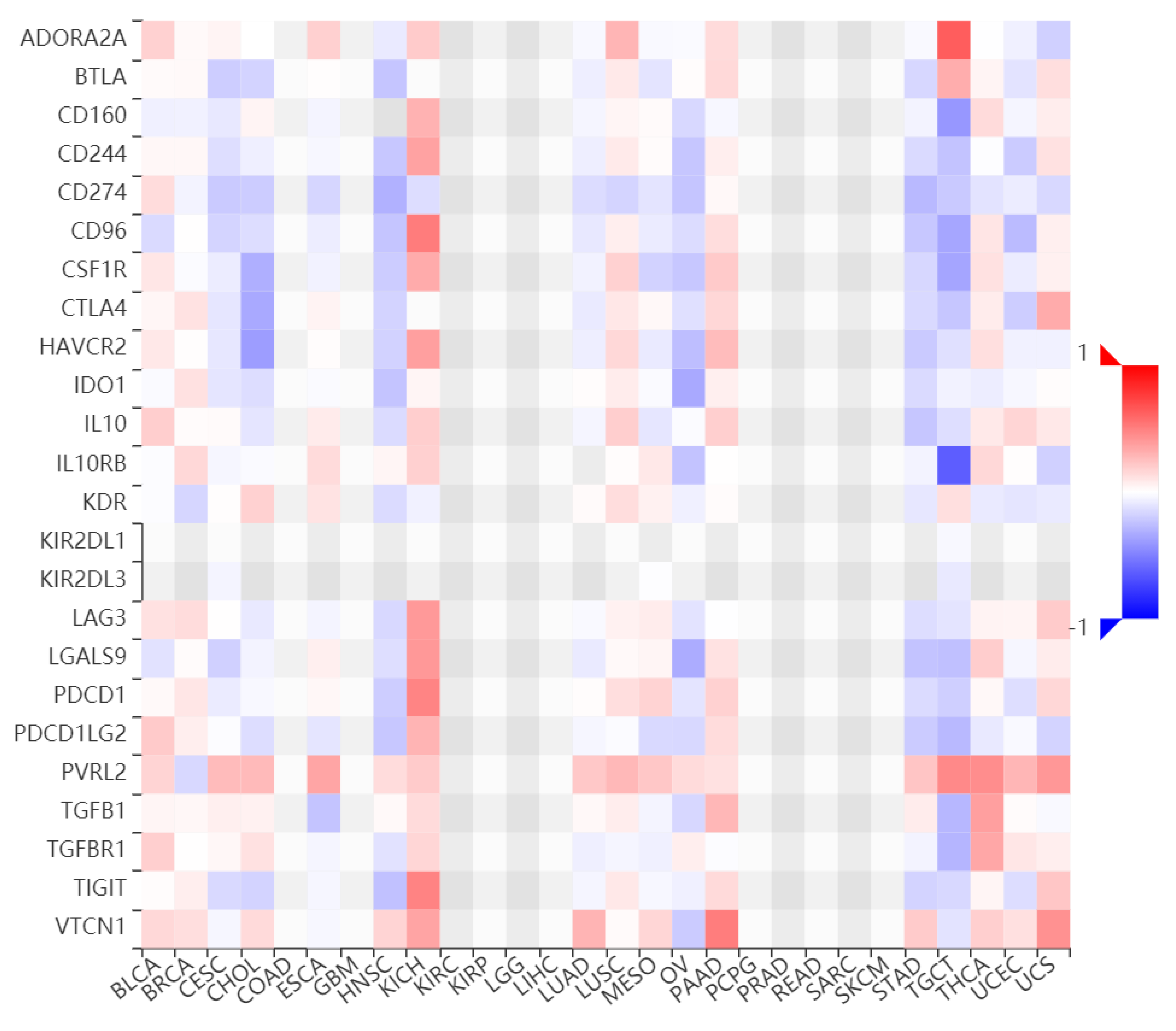

Supplement: Supplementary file 14 [file Image_2.TIF]
